# A novel fluorescent protein from the deep-sea anemone *Cribrinopsis japonica* (Anthozoa: Actiniaria)

**DOI:** 10.1038/srep23493

**Published:** 2016-03-22

**Authors:** Kenta Tsutsui, Eriko Shimada, Tomohisa Ogawa, Yusuke Tsuruwaka

**Affiliations:** 1 Department of Life and Environmental System Science, Graduate School of Nanobioscience, Yokohama City University, Yokohama, Kanagawa, Japan; 2 Japan Agency for Marine-Earth Science and Technology (JAMSTEC), Yokosuka, Kanagawa, Japan

## Abstract

A fluorescent protein was identified and cloned from the deep-sea anemone *Cribrinopsis japonica*. Bioluminescence and fluorescence expression were examined by direct observations of live specimens and RNA-Seq analysis. Both approaches revealed a novel green fluorescent protein in the tentacles of the anemone, but bioluminescence was not observed. Behavioural observations revealed that a blue light excited the fluorescence in the tentacles, and initiated a behavioural response whereby the fluorescent tentacles became fully exposed to the blue light. The excitation and emission peaks of *C. japonica*’s fluorescent protein were at 500 and 510 nm, respectively, which were greener than those reported in homologs. Furthermore, this protein was highly tolerant of increased temperatures and repeated freeze–thaw treatments. The current study presents an example of fluorescence in a deep-sea cnidarian, demonstrating that fluorescent proteins could have important roles, regardless of the presence or absence of strong sunlight. It also demonstrates that this deep-sea fluorescent protein has unique characteristics, including high stability, perhaps as an adaptation to the extreme environment.

The deep-sea is a dark, cold environment with high hydrostatic pressure. As sunlight is absorbed quickly by sea water, neither light nor heat reach great depths. However, it is now known that light is present in deep waters, via organisms producing light known as bioluminescence which is used for predation, defence, and communication^1^. With technological advances, it is now possible to observe bioluminescence in the deep-sea; these *in situ* observations have frequently witnessed bioluminescence generated by current-driven plankton colliding with structures on the sea floor^2^,^3^. The deep-sea, therefore, may not be as dark as scientists used to believe.

The sea anemone *Cribrinopsis japonica* Tsutsui & Tsuruwaka, 2014 is photosensitive, perhaps as a reflection of the abundance of light in the deep-sea. Surprisingly, this eyeless animal exhibited behavioural changes when exposed to blue light^4^, such as expansion of the oral disc and tentacles. More interestingly, the expanded tentacles emitted fluorescence, when excited by the blue light. Thus the question arises, why does the anemone have fluorescence in the deep-sea? Our knowledge of fluorescent proteins continues to expand. Previously, these proteins were found predominantly in shallow-water cnidarians^5^,^6^,^7^,^8^, and consequently the biological functions of these proteins have been considered as protection from intense sunlight and enhancement of photosynthesis^9^,^10^,^11^. However, we now know that fluorescent proteins are found in organisms without photosynthetic symbionts, including cnidarians^12^, amphioxus^13^ and deep-sea copepod^14^. Therefore, they may be expressed regardless of the presence or absence of photosynthesis. Recently, fluorescence has been observed in the deep-sea anemone *C. japonica*. Thus, the characterization of a deep-sea fluorescent protein may help deepen our knowledge of fluorescent proteins. Furthermore, it is interesting to investigate the evolution of fluorescent proteins in the deep-sea environment. Hence, this study identified, cloned and characterized fluorescent protein from the deep-sea anemone *C. japonica*.

## Results

### Fluorescence expression in *C. japonica*

**Figure 1:**
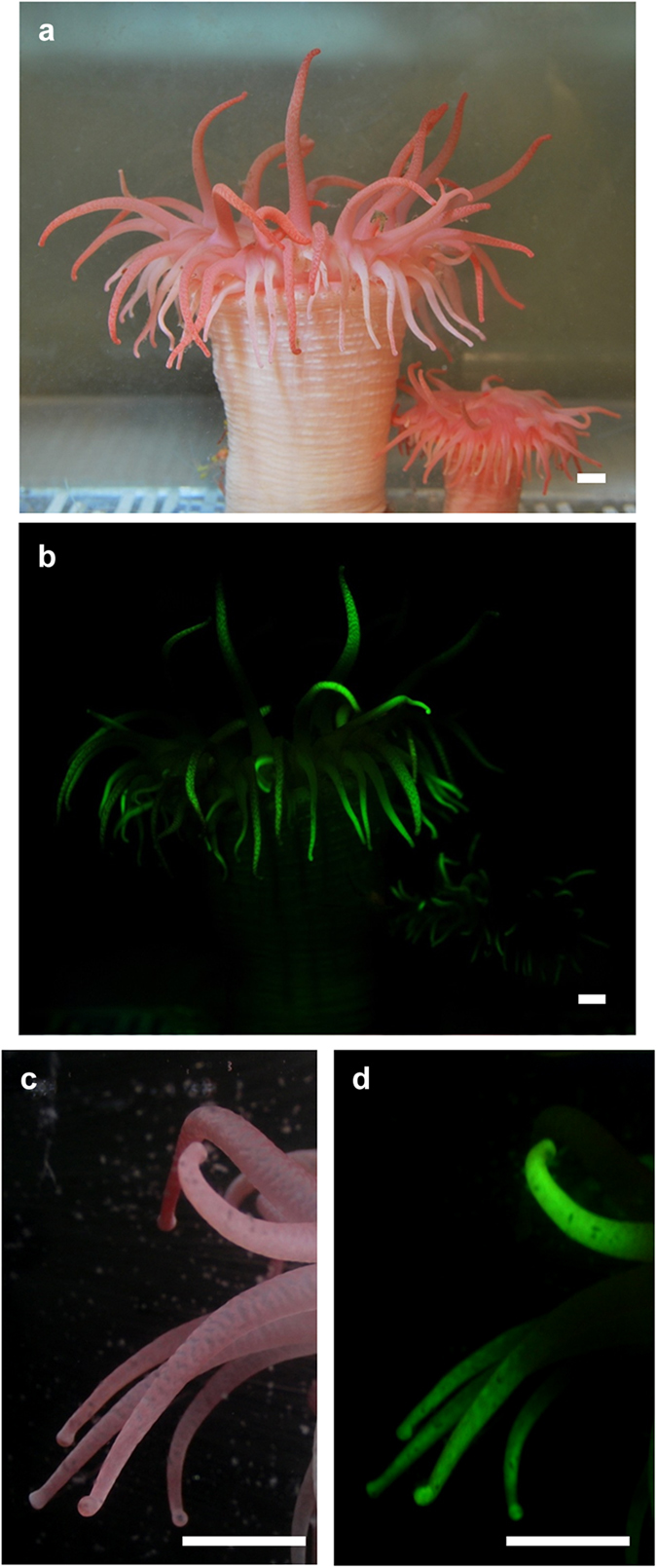
Fluorescence in *Cribrinopsis japonica.* (**a**) Under a fluorescent lamp (**b**) Through the cut-off filter under blue LED light, (**c**) Tentacles under a fluorescent lamp, (**d**) Tentacles observed through the filter under blue light. Scale bar: 1 cm.

**Figure 2:**
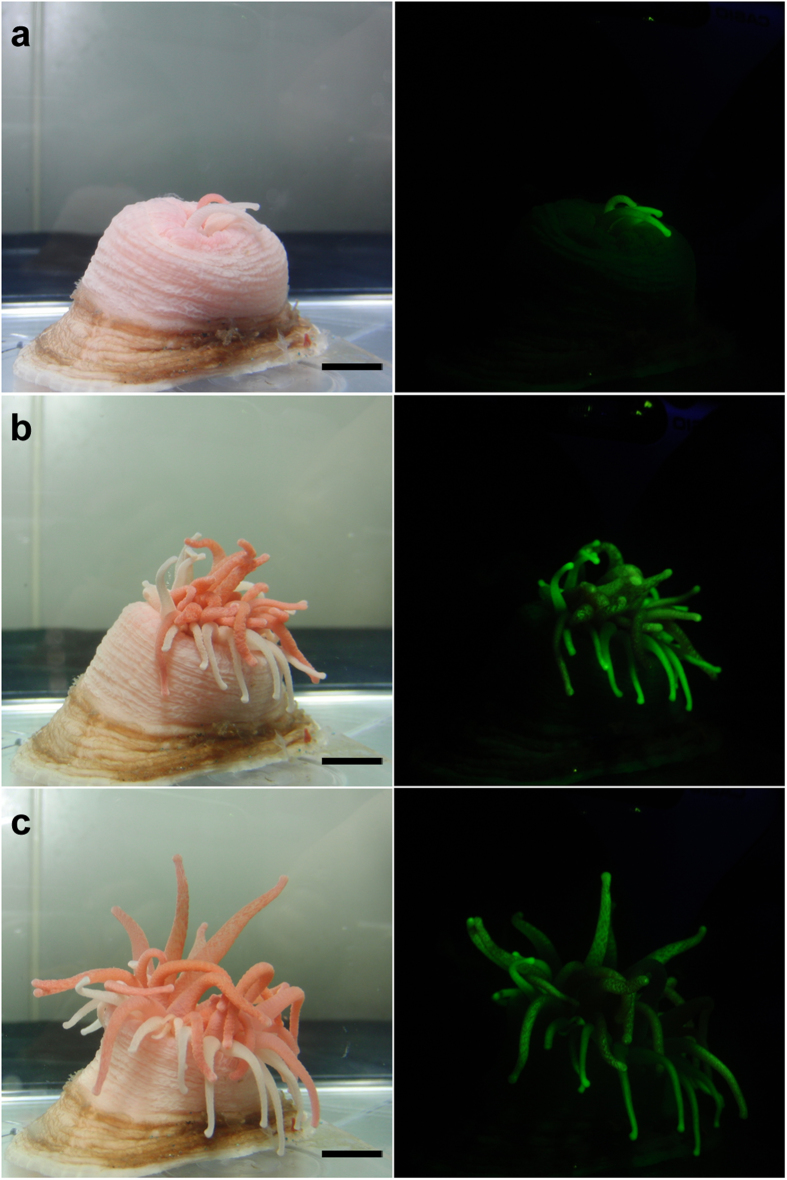
Increase in the fluorescence-expressing area of *Cribrinopsis japonica* after blue-light exposure. (**a**) 0 min after exposure, (**b**) 5 min after, (**c**) 10 min after. Scale bar: 1 cm. Photographs in the left column were taken without the cut-off filter under the fluorescent lamp, which was turned on only during photographing. The photographs in the right column were taken through the cut-off filter under blue LED light.

*Cribrinopsis japonica* is a pink anemone under fluorescent light (Fig. 1a). Bioluminescence has never been observed in this anemone. Physical stimuli, such as poking with a glass rod and massaging the anemone, induced the secretion of mucus and internal fluids, but these secretions were not luminous. When the anemone was exposed to blue light, green fluorescence was observed (Fig. 1b). The green fluorescence was observed only in the tentacles and was more intense at the tips (Fig. 1c,d). Consistent with the findings of a previous study^4^, the blue light caused the anemone to expand its oral disc and fluorescent tentacles. As the result, all the fluorescent tentacles were fully exposed to the blue light (Fig. 2).

**Figure 3:**
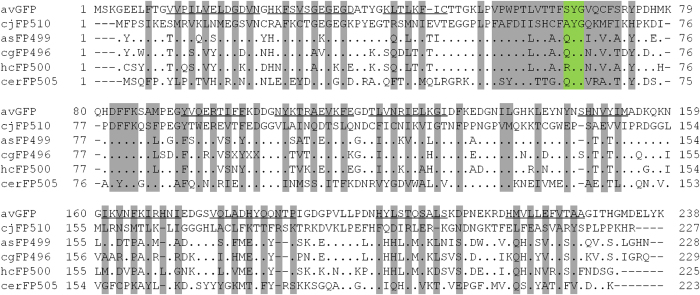
Primary structures of fluorescent proteins. Residues identical to those of cjFP510 are represented by dots and gaps are represented by dashes. avGFP: derived from *Aequorea victoria*, cjFP510: derived from *Cribrinopsis japonica*, asFP499: derived from *Anemonia sulcata*, cgFP496: derived from *Condylactis gigantea*, hcFP500: derived from *Heteractis crispa*, cerFP505: derived from *Cerianthus* sp. The peptide regions forming β-sheets in avGFP^29^ are underlined. Fluorophores are shaded in green, and the residues with the side chains that are presumably facing inward are shaded in grey.

Consistent with the lack of bioluminescence in the visual observation of live specimens, RNA-Seq did not yield sequences that were similar to previously known aequorin or luciferase. In contrast, the analysis indicated a sequence of 684 bp, which had high similarity with the actiniarian-origin fluorescent proteins (Table 1). The fluorescent protein derived from *C. japonica* (referred to as cjFP510 below) had the highest sequence similarity with asFP499 from *Anemonia sulcata* (identity = 66%), followed by cgFP496 from *Condylactis gigantea* (59%) and hcFP500 from *Heteractis crispa* (58%). The fluorophore (Ala63-Tyr64-Gly65) is typical and was similar to the homologs (Fig. 3). The sequence similarity between cjFP510 and cerFP505, which originated from a deep-sea tube anemone, was 35%.

**Figure 4:**
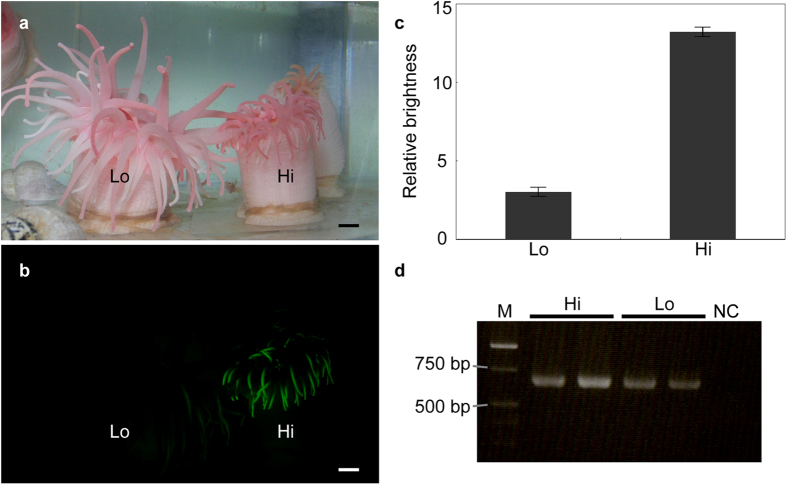
Highly fluorescent (Hi) and weakly fluorescent (Lo) *C. japonica.* (**a**) Anemones under the fluorescent lamp. (**b**) Fluorescence observed through the cut-off filter under blue LED light. (**c**) The brightness of the tentacles relative to the background ( = non-fluorescent area). (**d**) RT-PCR products. M: DNA marker, Hi: Highly fluorescent specimens (*n* = 2), Lo: Weakly fluorescent specimens (*n* = 2), NC: Negative control (dH_2_O). Scale bar: 1 cm.

The intensity of the fluorescence varied greatly among individuals (Fig. 4a,b), even though the specimens were maintained in the same aquarium under the same rearing conditions. There was no difference in coloration between the highly and weakly fluorescent individuals under the fluorescent lamp (Fig. 4a). The present study did not measure the fluorescence intensity of the live specimens directly. Instead, a bioimaging technique using ImageJ allowed us to measure the ‘brightness’ of the tentacles (Fig. 4b). The average brightness of the tentacles, relative to the background (i.e., non-fluorescent area in the figure), was 3.0 (*n* = 28; S.D. = 1.62) for weakly fluorescent specimens and 13.2 (*n* = 26; S.D. = 1.40) for highly fluorescent ones (Fig. 4c). From RT-PCR of the cjFP510 gene in total RNA, bands of approximately 700 bp were observed in both highly and weakly fluorescent specimens of *C. japonica* (Fig. 4d), consistent with the size suggested by RNA-Seq. Sequence analysis of these amplified products revealed that despite the varying fluorescence, the products were identical (data not shown).

### Recombinant fluorescent protein

**Figure 5:**
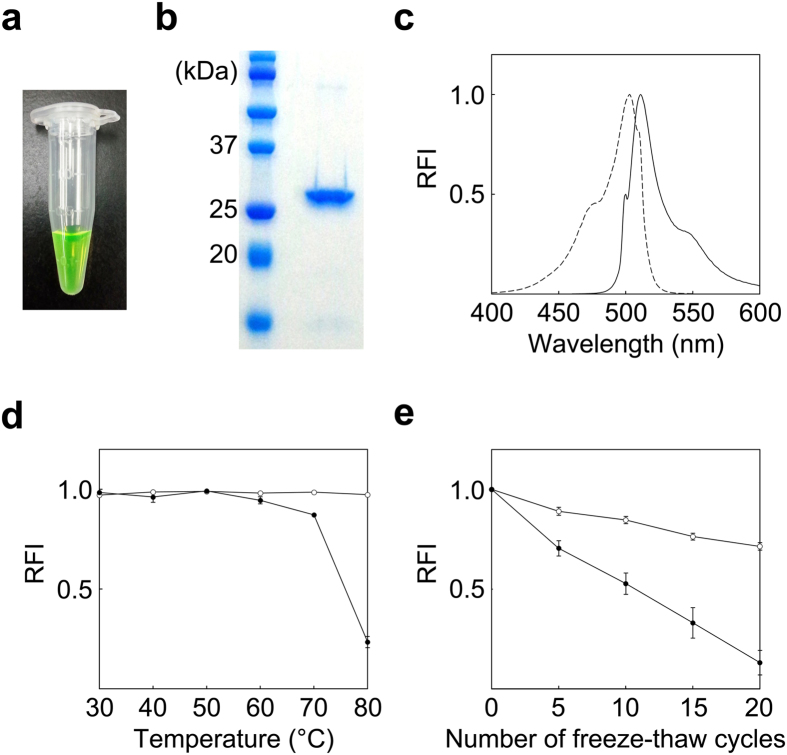
Recombinant cjFP510 and its stability. (**a**) Recombinant cjFP510 purified from the bacterial cell. (**b**) SDS-PAGE of purified recombinant cjFP510. (**c**) Excitation (---) and emission (—) spectra. (**d**) Tolerance of cjFP510 (○) and EmGFP (•) to high temperatures. (**e**) Tolerance to freeze–thaw cycles. In both stability experiments, 0.1 μg/μL of cjFP510 or EmGFP was prepared in 10 mM phosphate buffer (pH 7.4). RFI: Relative fluorescence intensity. Vertical bars represent the standard errors.

Expression of His-tagged recombinant cjFP510 (Fig. 5a) was performed successfully using *Escherichia coli*, BL21 (DE3). Functional cjFP510 was produced by inducing expression at 16 °C for 40 h. Induction of the protein at higher temperatures (>30 °C) dramatically decreased the fluorescence intensity of the bacterial cell. Purified cjFP510 had a molecular weight of approximately 26 kDa (Fig. 5b), which was consistent with the predicted weight of 26.7 kDa, known from the primary structure of the protein. The peak excitation and emission spectra were 500 and 510 nm, respectively (Fig. 5c).

### Stability

To determine whether cjFP510 was adapted to extreme conditions (e.g., high hydrostatic pressure, low temperature) in the deep-sea, this study evaluated the stability of recombinant cjFP510, in terms of its tolerance to increased temperatures and repeated freeze-thaw cycles. One of the most remarkable characteristics of cjFP510 was its thermotolerance; it maintained its maximum fluorescence intensity even after incubation at 80 °C for 1 h (Fig. 5d; Table 2). In comparison, the fluorescence of Emerald GFP was just 23% of its maximum intensity after the same treatment. The high stability of cjFP510 was also supported by its tolerance to repeated freeze–thaw cycles. As shown in Fig. 5e and Table 3, cjFP510 retained approximately 70% of its maximum intensity after 20 freeze–thaw cycles, whereas the fluorescence intensity of EmGFP was 53% of its maximum after 10 cycles and 13% after 20 cycles.

## Discussion

To the best of our knowledge, this is the second report of a fluorescent protein identified from a deep-sea cnidarian, following cerFP505 that was isolated from a deep-sea ceriantharian^15^. However, there was little similarity between the primary structures of these two deep-sea fluorescent proteins. The amino acid sequence of cjFP510 was quite similar to those identified in shallow-water actiniarians (Fig. 3). Labas *et al*.^7^ grouped these shallow-water proteins into one clade consisting exclusively of actiniarian proteins, showing that the clade was distinct from other non-actiniarian fluorescent proteins. The low similarity between cjFP510 and cerFP505, both from the deep-sea but taxonomically distant, might indicate that the evolution of fluorescent proteins was influenced more strongly by the evolution of the individual species rather than the environment.

Despite the close relationship between cjFP510 and the shallow-water actiniarian fluorescent proteins, the long excitation spectrum of cjFP510 makes the protein distinct from others. The excitation peak of cjFP510 (500 nm) was longer than that of asFP499, cgFP496, and hcFP500, whose excitation peaks were 403/480, 399/482, and 405/481 nm. Johnsen *et al*.^3^ reported that the wavelengths of deep-sea bioluminescence tended to shift towards green, so the long excitation peak of cjFP510 may be an adaptation to deep-sea greenish bioluminescence, which could be the excitation light source.

Another remarkable feature of cjFP510 was its high stability. The current study indicated that cjFP510 exhibited higher tolerance to increased temperatures and repetitive freeze-thaw cycles than the avGFP-derivative Emerald GFP. Furthermore, cjFP510 was more stable than other fluorescent proteins; a considerable reduction in fluorescence intensity was observed after incubation at 80 °C for 5 min in wild-type avGFP^16^ and in asFP499^17^, the closest homolog of cjFP510. This high stability of cjFP510 may not be an adaptation to low temperatures in the deep-sea, because cold-adapted enzymes tend to be more flexible but less thermotolerant than meso- and thermophilic enzymes^18^, inconsistent with the features of cjFP510. It should be noted, however, that a fluorescent protein is not considered an enzyme, so low temperatures may affect the protein differently. The deep-sea is also characterized by high hydrostatic pressure. Although increased pressure dissociates oligomerized proteins and interferes with their functions^19^, pressures below 400 MPa generally do not denature a monomeric protein (reviewed in Gross and Jaenicke^20^). It remains unclear whether the high stability of cjFP510 resulted from an adaptation to the deep-sea. However, a similar adaptation was seen in deep-sea fish, where lactate dehydrogenase from these fish has a higher tolerance to high pressure and increased temperature compared with shallow-water fish^21^. Additionally, β-agarase from the bacterial strain isolated from the deep-sea sediment was thermostable up to 60 °C^22^. A single environmental factor alone may not explain the high stability of cjFP510, but a combination of the multiple deep-sea factors could favour proteins with high stability in the deep-sea.

The biological role of cjFP510 in this deep-sea anemone has not been explained satisfactorily in previous studies. In addition to photoprotection and photosynthesis enhancement, fluorescent proteins have also been known to react with free radicals^23^ and, thus, have the potential to act as antioxidants in living animals. However, localization of concentrated cjFP510 in tentacles makes their role as antioxidants unlikely in *C. japonica*. Additionally, high levels of oxygen in the tissues of *C. japonica* are unlikely in the deep-sea, whereas oxygen is highly concentrated in the tissues of shallow-water corals because of photosynthesis^24^.

**Figure 6:**
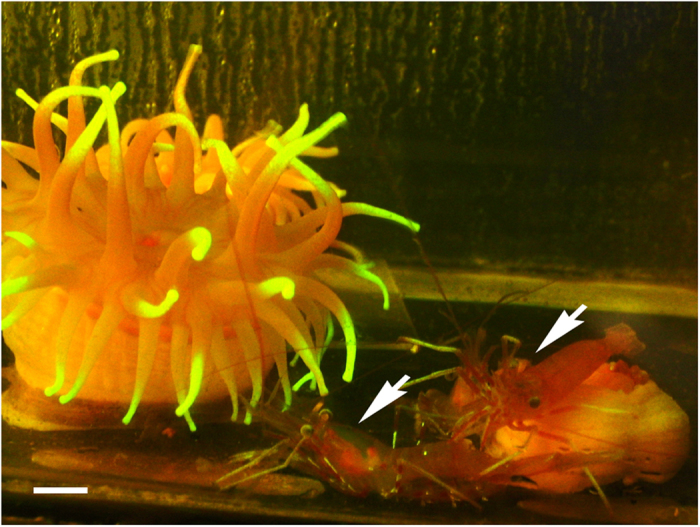
*Cribrinopsis japonica* and the deep-sea shrimp in the laboratory. The photograph was taken through the cut-off filter under fluorescent lamp and t blue LED light. Arrows indicate the shrimp. Scale bar: 1 cm.

Another important biological role of fluorescent proteins is prey attraction. There is experimental evidence from Haddock and Dunn^25^, who observed juvenile rockfish attacking the fluorescent tentacles of the hydromedusa *Olindias formosus*, and concluded that fluorescence can be a strong attractant to visual predators. In the case of *C. japonica*, the anemone is usually surrounded by a co-living shrimp under laboratory conditions (Fig. 6) and often captured along with these shrimp at the depth of 800 m^26^. Hence, the anemone probably attracts the shrimp via visual or chemical cues. In addition, the previous study^4^ discussed that the light-induced expansion behaviour of *C. japonica* appeared similar to the preparatory feeding behaviour of *Urticina felina* (described in detail by McFarlane^27^), so the glowing tentacles of *C. japonica* could function as a lure for prey attraction. Although the findings of this study are not sufficient to confirm that fluorescence of the anemone indeed attracts shrimp or prey, the idea of the potential role of fluorescence as an attractant should not be discarded.

Finally, variations in fluorescence intensity in *C. japonica* may be useful for determining the importance of the fluorescent protein in the deep-sea. All specimens used in this study were maintained in the same aquarium under the same rearing conditions. Thus, environmental factors such as temperature, salinity, and nutrition could not explain the variation in expression, which must be due to intrinsic factors such as age, sex, and mutations in gene regulators. If expression of fluorescence was indeed beneficial in the deep-sea, there would be a biased population ratio of highly fluorescent to weakly fluorescent individuals *in situ*. Therefore, monitoring the population of the two groups of *C. japonica in situ* should help us evaluate the importance of fluorescence in the deep-sea.

As knowledge of the presence of fluorescent proteins in deep waters is relatively new, many questions remain unanswered. What is the excitation light? Is high stability unique to deep-sea fluorescent proteins? Do fluorescent proteins have a significant function related to capturing a prey or venom other than emitting light in the dark environment? To answer these questions, discoveries and analyses of other deep-sea fluorescent proteins will be helpful. We hope this report will encourage researchers to pay more attention to deep-sea fluorescent proteins and accelerate further research in the future.

## Materials and Methods

### Specimens

Specimens of the deep-sea anemone *Cribrinopsis japonica* were collected at a depth of 600 m in Toyama Bay, Sea of Japan, as described in Tsuruwaka and Shimada^28^. Specimens were maintained in artificial seawater at 2 °C in the dark. About one third of the aquarium water was replaced every week to keep the anemones healthy. Dried krill was given to the anemone once a week.

### Examination of fluorescence and bioluminescence

The fluorescence in *C. japonica* was observed through a < 520-nm cut-off filter (Fujifilm, Tokyo, Japan), which blocked wavelengths shorter than 520 nm, under a blue LED light of 470 nm (OptoCode, Tokyo, Japan). The anemone was not bioluminescent. Some deep-sea anemones, however, were seen to produce luminous secretions^3^, so the present study was interested in whether the biological fluid secreted from *C. japonica* was luminous. Secretion of the biological fluid was promoted by poking the anemone with a glass rod and massaging it gently in darkness.

Fluorescence intensity in live specimens was measured using ImageJ (the National Institutes of Health, USA). A photograph of both highly and weakly fluorescent specimens was first converted into grey scale by the “split channels” command. The green-channel image showed fluorescent tentacles clearly, so it was selected for further analysis. A whole tentacle (from the base to the tip) was encircled, whenever possible, and the maximum brightness within the encircled area was measured. In a highly fluorescent specimen, 26 encircled areas were measured, with 28 areas circled in a weakly fluorescent specimen. Twenty separate background areas were also randomly selected for measurements. Each brightness value measured from the tentacles was divided by the average maximum brightness of the background.

A previous study indicated that *C. japonica* responded to blue light by expanding its oral disc and tentacles^4^. A change in the fluorescence-expressing area associated with photoresponsive behaviour was observed under blue LED light. The anemone was placed in an aquarium filled with fresh artificial seawater. After a 30-min acclimation period, the anemone was exposed to blue light for 10 min. Photographs were taken every 5 mins, first under blue light and subsequently under a fluorescent lamp with the blue light off. The cut-off filter was only used during photographing under blue light.

### RNA-Seq

Total RNA was extracted from the tip of a tentacle of *C. japonica*. After synthesis of cDNA using the oligo dT primer, RNA-Seq was performed using Hiseq 2000 (Illumina, San Diego, CA, USA). Assembled sequences were run through the BLAST program to identify known photoproteins, such as aequorin and luciferase, and fluorescent proteins.

### Cloning of fluorescent protein

Total RNA was isolated from *C. japonica,* and subsequently cDNA was synthesized. Based on the obtained sequence of a fluorescent protein from RNA-Seq, RT-PCR was carried out using the primers: 5′-ATGTTTCCCTCGATCAAAGAAAGC-3′ (forward) and 5′-TTAACGGTGTTTGGGAGGCAAG-3′ (reverse). The amplified gene fragment was then ligated in the pTAC-2 vector (BioDynamics Laboratory, Tokyo, Japan). The constructed plasmid, labelled as pTAC2-cjFP510, was cloned in a bacterial cell and then sequenced to ensure consistency between the insert and the sequence obtained from RNA-Seq. The obtained gene sequence is available in the DDBJ Sequenced Read Archive under the accession number LC123595.

For expression in *Escherichia coli*, the cjFP510 gene was amplified using pTAC2-cjFP510 as the template and the primers that contained either the *Nde* I or *Xho* I digestion site: 5′-TAGTCATGCATATGTTTCCCTCGATCAAAG-3′ (forward, *Nde* I) and 5′-GGAACTCGAGACGGTGTTTGGGAGGC-3′ (reverse, *Xho* I). The amplicon was digested with *Nde* I and *Xho* I and ligated in the pET22b(+) vector (Novagen). As there was another *Nde* I digestion site within the gene, the reaction time for *Nde* I was shortened from 120 to 5 min. After electrophoresis, two bands (≈700 bp, ≈600 bp) were observed, and the ≈700 bp band was purified and used for ligation. The resulting plasmid was sequenced to confirm the absence of mutations.

### Preparation of Emerald GFP (EmGFP)

Emerald GFP was amplified using pRSET-EmGFP (Invitrogen) as a template and the following primers: 5′-AAGAGCATATGGTGAGCAAGGGCGAGGAGCTG-3′ (forward, *Nde* I) and 5′-TTGAACTCGAGCTTGTACAGCTCGTCCATGCCGAG-3′ (reverse, *Xho* I). The amplicon was treated with restriction enzymes *Nde* I and *Xho* I and subsequently ligated in the pET22b(+) vector.

### Expression of recombinant proteins in E. coli

BL21 DE3 was transformed with the constructed plasmid, either pET22b + cjFP510 or pET22b + EmGFP. After formation of colonies on the LB agar plates containing ampicillin (100 μg/ml), a single colony was transferred to ampicillin-containing liquid culture and incubated at 37 °C until the OD_600_ reached 0.7–0.9. Then, the fluorescent protein was induced at a temperature of 16 °C for 40 h with 0.5 mM IPTG for cjFP510 and at 37 °C for 5 h with 0.5 mM IPTG for EmGFP. The cultured cells were collected by centrifugation, rinsed once, resuspended with the binding buffer (20 mM Phosphate, 500 mM NaCl, 40 mM Imidazole, pH 7.4), and then lysed by sonication. Purification of the protein was done using the polyhistidine-tag. The protein was eluted with the elution buffer (20 mM Phosphate, 500 mM NaCl, 500 mM Imidazole, pH 7.4). To remove salt and imidazole, the buffer was replaced with 10 mM phosphate buffer (pH 7.4) using the centrifuge filter. The excitation and emission spectra of the purified recombinant protein were measured using FluoroMax-4 (Horiba, Kyoto, Japan).

### Stability analyses

To measure the thermostability of cjFP510, 0.1 μg/μL of the recombinant protein was incubated at 30, 40, 50, 60, 70, or 80 °C for 1 h. Additionally, to determine the effect of repeated freeze–thaw cycles on the recombinant cjFP510, 0.1 μg/μL of the recombinant protein was frozen at 80 °C for at least 30 min and then thawed at 25 °C. This cycle was repeated 20 times. The fluorescence intensity after the treatments described above was measured using Synergy HT (BioTek, Winooski, VT, USA) at an excitation wavelength of 485 nm and an emission wavelength of 528 nm. The same treatments were performed for EmGFP.

## Additional Information

**How to cite this article**: Tsutsui, K. *et al*. A novel fluorescent protein from the deep-sea anemone *Cribrinopsis japonica* (Anthozoa: Actiniaria). *Sci. Rep.* **6**, 23493; doi: 10.1038/srep23493 (2016).

## Figures and Tables

**Table 1:** Actiniarian fluorescent proteins and a deep-sea ceriantharian fluorescent protein.

FP*	Organism (Class/Order)	Identity to cjFP510	Excitation (nm)	Emission (nm)	Reference
cjFP510	*Cribrinopsis japonica* (Anthozoa/Actiniaria)	–	500	510	Present work
asFP499	*Anemonia sulcata* (Anthozoa/Actiniaria)	66%	403/480	499	a
cgFP496 (cgigGFP)	*Condylactis gigantean* (Anthozoa/Actiniaria)	59%	399/482	496	b
hcFP500 (hcrGFP)	*Heteractis crispa* (Anthozoa/Actiniaria)	58%	405/481	500	b
cerFP505	Ceriantharian (Anthozoa/Ceriantharia)	35%	494	505	c

^*^The original name for the protein is indicated in parentheses. References: a, Wiedenmann *et al*.^17^; b, Labas *et al*.^7^; c, Vogt *et al*.^15^.

**Table 2:** Thermostability in two fluorescent proteins.

	Temperatures (°C)					
	30	40	50	60	70	80
cjFP510	0.971 ± 0.02	0.987 ± 0.02	0.989 ± 0.01	0.981 ± 0.01	0.986 ± 0.01	0.973 ± 0.02
EmGFP	0.984 ± 0.01	0.961 ± 0.03	0.992 ± 0.01	0.945 ± 0.02	0.872 ± 0.01	0.235 ± 0.03

Relative fluorescence intensity (Average ± S.D.) is shown. Highest intensity was defined as 1. Experiments were conducted on four independent occasions.

**Table 3:** Tolerance to repeated freeze–thaw cycles.

	Number of freeze–thaw cycles				
	0	5	10	15	20
cjFP510	1	0.890 ± 0.04	0.847 ± 0.04	0.763 ± 0.04	0.714 ± 0.04
EmGFP	1	0.705 ± 0.04	0.528 ± 0.05	0.331 ± 0.08	0.132 ± 0.06

Relative fluorescence intensity (Average ± S.D.) is shown. The intensity of the control sample was defined as 1. Experiments were conducted on four independent occasions.
